# Patient cost analysis of a community-based teledermatology service versus conventional outpatient appointments in East Kent: a retrospective study through a societal lens to reduce health inequalities

**DOI:** 10.1186/s12913-024-12112-7

**Published:** 2024-12-21

**Authors:** Nurul Ain Nizar, Roopa Farooki, Piyush Mahapatra, Saul Halpern, Tim C. H. Hoogenboom

**Affiliations:** 1 Open Medical Ltd, London, UK; 2 Dermatology Department, Kent and Canterbury Hospital, Ethelbert Road, Canterbury, Kent, CT1 3NG UK

**Keywords:** Teledermatology, Health services accessibility, Health inequities, Cost analysis, Community health services

## Abstract

**Background:**

The UK's National Health Service (NHS) is grappling with rising demand and limited dermatologists, leading to longer waiting times. This is particularly concerning for conditions like malignant melanoma, where early diagnosis is crucial. Teledermatology is being introduced to address these issues, but its impact on patients’ monetary and time costs, especially in deprived areas, is under-researched. This study investigates the impact of a community-based teledermatology model in East Kent, a coastal region with high cross-regional health inequalities.

**Methods:**

We analysed the financial costs and time invested by patients undergoing community-based teledermatology versus if they were to attend traditional appointments. Data were gathered from 1368 referrals, from May 2022 to January 2024 at a secondary care provider in the region. We considered the diagnosis method, appointments, travel, monetary cost of time, parking costs and Index of Multiple Deprivation Deciles (IMDD).

**Results:**

Our study showed the community teledermatology model significantly reduced the active patient’s time and cost to obtain a diagnosis. Average time was 38.1 min, compared to 96.7 min in traditional clinics, saving 58.4 min (95% CI -62.3 to -54.5, *p* < 0.001). Patients saved £17.9 per diagnosis with this model (95% CI -£19.2 to -£16.6, *p* < 0.001). Time savings were more substantial for patients from more deprived regions (IMDD 1–3).

**Conclusion:**

The community-based teledermatology model proves to be cheaper for patients, providing a convenient alternative to traditional clinics, particularly benefiting patients from deprived backgrounds by improving healthcare accessibility and offering flexible consultation options.

**Supplementary Information:**

The online version contains supplementary material available at 10.1186/s12913-024-12112-7.

## Background

The United Kingdom's National Health Service (NHS) is facing a significant challenge in providing adequate dermatology services [1].

A rising demand for outpatient appointments and limited number of dermatologists is leading to an alarming increase in waiting times, thereby increasing the potential for patients' conditions to worsen [2]. In the context of malignant melanoma, where early diagnosis is key to achieve curative treatment, this development is particularly concerning. According to the national report Get It Right First Time (GIRFT), there were approximately 3.5 million outpatient and day surgery visits between 2018 and 2019, while the NHS only had 659 consultant dermatologists available to handle this workload [2].

Alongside the extended waiting period, patients are also faced with considerable financial costs and time investment [3, 4], especially in rural and coastal regions. This situation exacerbates health disparities as these costs disproportionately impact individuals from disadvantaged backgrounds and has worsened in the recent cost-of-living crisis.

Teledermatology is increasingly introduced to address workforce constraints, reduce waiting times for skin cancer referrals, and demonstrate further benefits such as improved healthcare access and higher patient satisfaction [5–7]. However, there is a lack of research focusing on the impact of such services on ameliorating the costs for individual patients, especially when employed in areas experiencing high deprivation characterised by complex socio-economic challenges.

This research examines the impact of a teledermatology model that introduced community-based dermoscopy services into the patient care pathway (Pathpoint eDerma, Open Medical ltd) on both the financial and time cost for individual patients referred for skin lesion assessment in the East Kent region. This region, which includes several coastal towns, experiences poorer health outcomes and higher rates of premature death compared to the national average. Specifically, the rates of COPD, depression, and diabetes are 48%, 28%, and 11.9% higher, respectively, than those in the rest of England. These higher disease rates are primarily due to the region's larger population of residents aged 55 and above [8, 9].

Additionally, the region faces socio-economic challenges that contribute to a high level of overall deprivation and worsening health inequalities. In terms of socio-economic indicators, 32.2% more people live in the most deprived neighbourhoods in terms of employment, 17.7% more in education, and 24.8% more in income levels in the coastal part of East Kent [8, 9]. The region also struggles with access to specialised services like dermatology and recruitment of healthcare and social care staff due to its remote areas [9]. The combination of an ageing population, severe deprivation, geographical barriers, and limited healthcare facilities demonstrated the need for the introduction of teledermatology services in the region.

The aim of this research is to assess the patient costs in the East Kent region, comparing the newly implemented teledermatology service with conventional face-to-face outpatient appointments, focusing on health inequalities. By investigating the monetary and time costs associated with these two care models, from the point of primary care referral to obtaining a diagnosis, this study aims to provide valuable insights into the societal implications of teledermatology services for patients in high-deprivation coastal areas.

## Methods

### Study setting

The dermatology department for East Kent Hospitals University NHS Foundation Trust (EKHUFT) is housed within the Kent and Canterbury Hospital, where the vast majority of outpatient appointments take place. In May 2022, a novel teledermatology service was introduced.

**Fig. 1:**
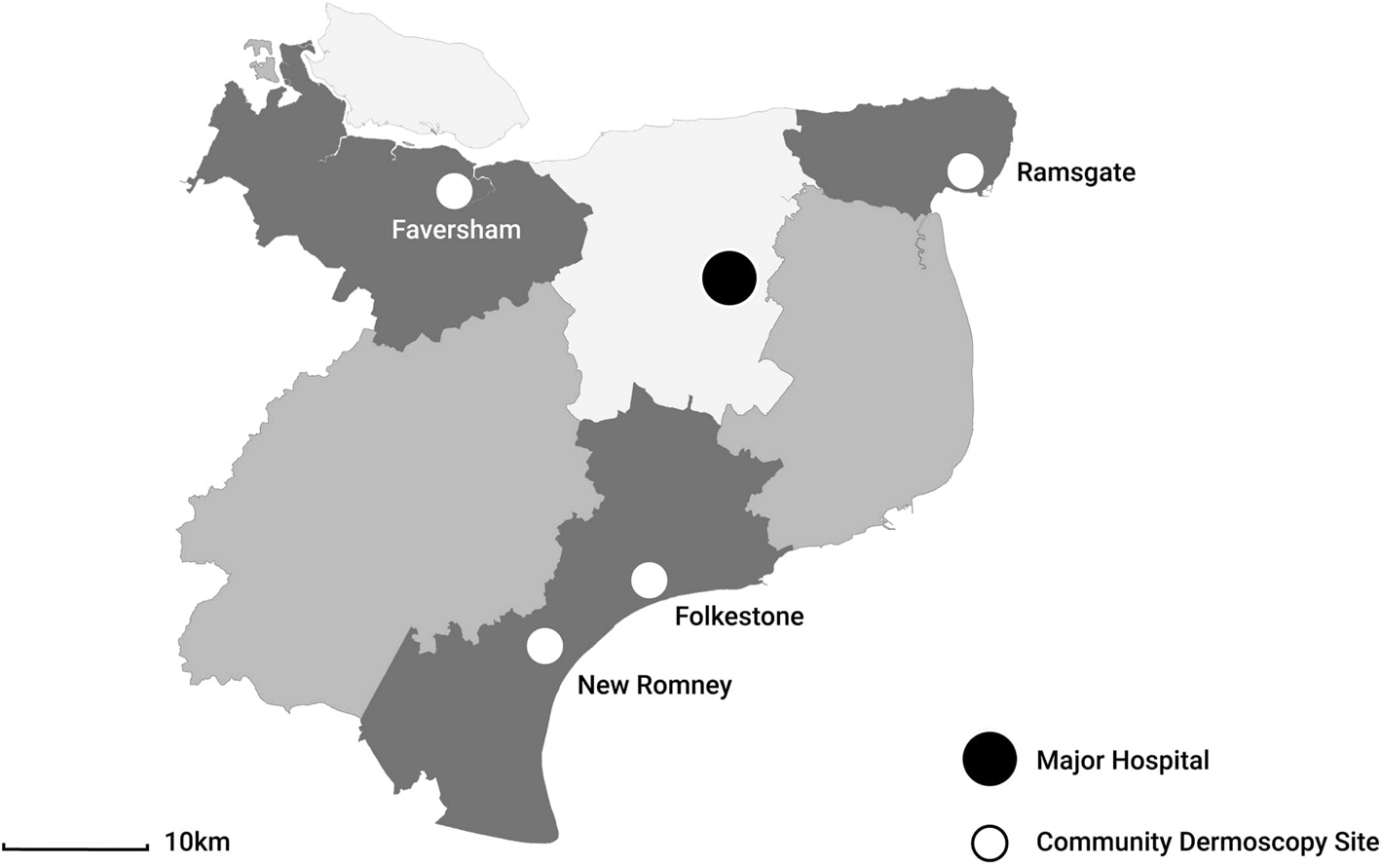
Location of CD sites and major hospital

As part of the implementation, four community-based dermoscopy services were opened in four locations where patients could have their skin lesions photographed by a healthcare assistant. These locations are Faversham, Ramsgate, Folkestone, and New Romney. These sites are strategically located in different coastal towns across the region, allowing patients to be referred to the site closest to their homes for dermoscopy appointments. The photographs of the lesions, along with a digitally completed patient questionnaire, are then uploaded into Pathpoint for remote assessment by a Consultant Dermatologist. Figure. 1 provides a visual representation of the geographical distribution of the community dermoscopy (CD) sites and the dermatology department.

**Fig. 2:**
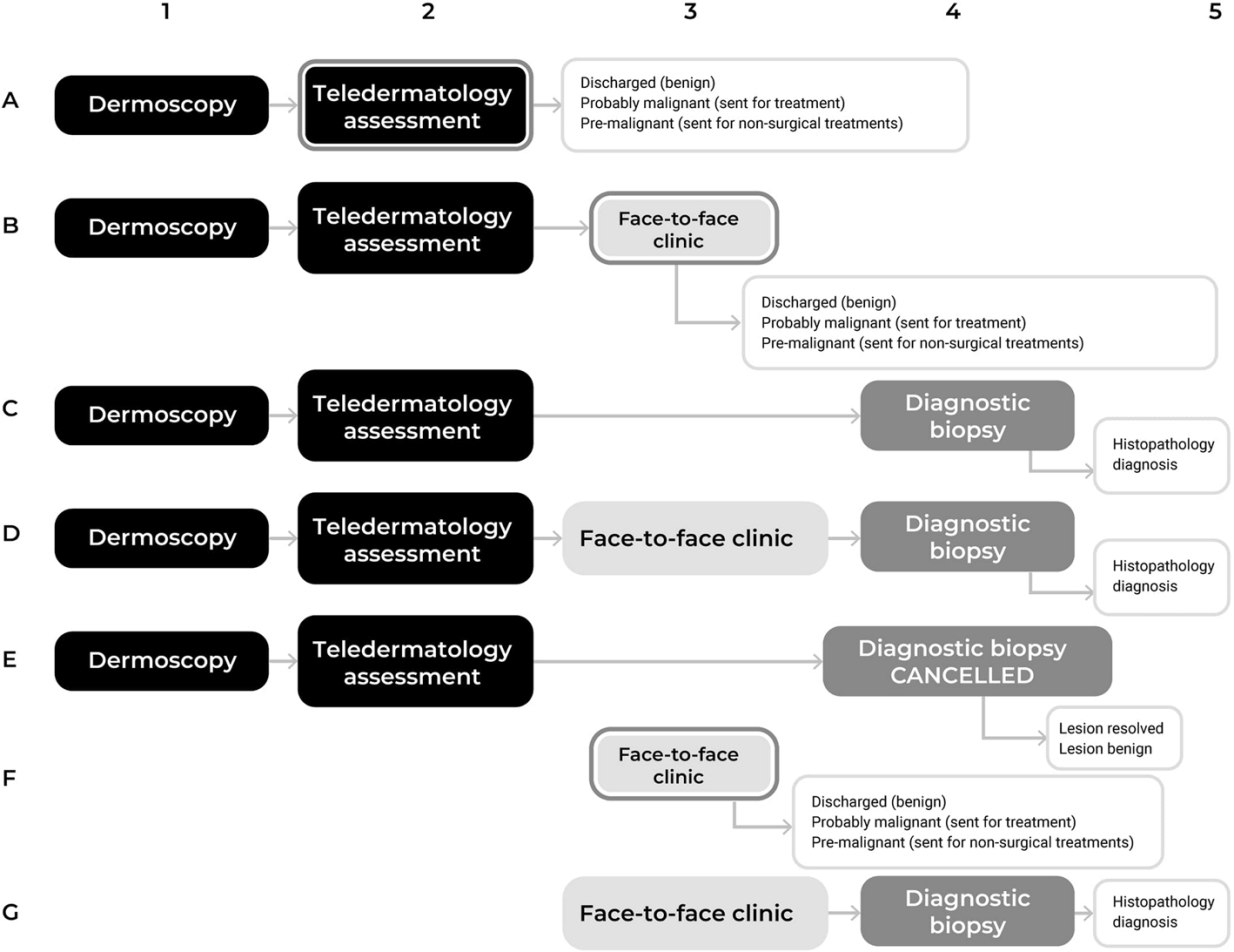
Patients’ journeys pathways

The patient's journey to either a conventional face-to-face outpatient appointment or the teledermatology service is simplified in Fig. 2. The teledermatology service offers five potential pathways (Pathway A, B, C, D, and E) for the patient's journey, contingent on the dermatologist's remote evaluation of the skin lesion images. In contrast, the traditional outpatient service typically follows either Pathway F or G. The possible outcomes for patients in both services range from discharge, referral for further treatments including excision or non-invasive, or a diagnostic biopsy to obtain a histopathological diagnosis for the skin lesion.

### Study design and outcome measures

We conducted a retrospective cost analysis, comparing the time and monetary expenses incurred by patients who attended teledermatology sessions with a hypothetical scenario where they would have attended traditional face-to-face outpatient appointments at the dermatology department.

### Participants

The data for this analysis were collected from May 2022 to January 2024. They represent patients referred for an urgent skin lesion assessment, where exclusion of malignancy is the primary aim. Notably, this is a different cohort than the ‘2 week-wait’ referral pathway, who are considered at higher risk. A total of 1368 referrals to the teledermatology service were included in the primary analysis of costs for patients. A Health Inequalities Survey (HIS) was distributed to patients digitally, which was completed by 614 patients. HIS can be found in the Supplementary File.

### Data collection

The project received ethical consent from The East Kent Hospitals University NHS Foundation Trust Clinical Audit and Improvement Team. As the project used anonymised data from standard care and optional questionnaires, it was classified as a clinical audit, negating the need for further ethical review (Reference Number: RN903073).

To calculate the costs for patients, we computed the total financial cost and time spent by patients from the initiation of referral to the point of receiving a diagnosis for their skin conditions. We adopted the methodology used in prior research to calculate the average cost of attending a teledermatology or in-person appointment [3, 10]. As with previous studies, we also referred to published national data to establish the appropriate values for our calculations. This approach was taken to improve the reliability of our study [3, 10]. The cost analysis considered the expenses associated with attending a dermoscopy appointment, an outpatient appointment, and undergoing a diagnostic biopsy procedure, depending on the individual patient pathways.

We sourced data from the digital clinical pathway, Pathpoint eDerma, and patient responses from the HIS. Additionally, we used publicly accessible data such as fuel costs for travel, parking fees at both the CD and hospital sites, the hourly wage rate to gauge the financial cost of time lost for working patients, the hourly rate of leisure time for non-working patients, and the average duration of outpatient, dermoscopy, and biopsy procedure appointments. We were able to calculate travel time based on individual patient distance to CD and hospital sites. A summary of these data and their sources can be found in Table 1 (page 23).

**Table 1:** Sources of data used in the analysis (page 7, line 19)

No	Data Item	Unit	Value and Year	Source
1.	Distance from patients’ homes (approximate) to CD and hospital sites. (cds_dist, hosp_dist)	kilometre	Value varies. The distance was calculated in 2024, at the point of data extraction	Pathpoint pgeocode
2.	Cost of petrol	£ per litre	2022: £1.758 2023: £1.608 2024: £1.568	Department for Energy Security and Net Zero, Monthly and annual prices of road fuels and petroleum products [11]
3.	Parking fee at CD sites	£ per visit	£1.45 for Whitstable, Ramsgate and New Romney £2.2 for Folkestone Due to the unavailability of cost data for a specific year, 2023 values were used, as they represent the vast majority of our cases: 2023: 1191 cases 2022: 175 cases 2024: 2	Publicly available data [12]
4.	Parking fee at hospital site	£ per visit Clinic/ Biopsy	£2.5/3 Due to the unavailability of cost data for a specific year, 2023 values were used, as they represent the vast majority of our cases	East Kent Hospitals University NHS Foundation Trust website [13]
5.	Average wage for working patients (used to calculate Monetary cost of time)	£ per hour	Value varies depending on annual income bracket reported in HIS. The average was calculated in 2024, at the point of data extraction	HIS
6.	Average cost of leisure time for non-working patients (used to calculate Monetary cost of time)	£ per hour	2022: £10.96 2023: £10.93 2024: £10.94	Department of Transport, Transport Analysis Guidance (TAG) Unit A1.1 November 2023 [14]

To understand the health inequalities affecting our patient population, we correlated patient demographic data and our findings with the national Index of Multiple Deprivation Decile (IMDD) [15].

#### Travel time calculation

The calculation of travel distance involved measuring the distance between the patient's reported postcode and both the dermoscopy and secondary care sites. This was achieved by using the pgeocode library (GeoDistance('gb')). To promote anonymity, a random shift in distance ranging from −5% to + 5% was incorporated. The type and number of trips were determined based on the method of diagnosis as outlined in Table 2. This facilitated the calculation of total kilometres driven for diagnosis. A regional average travel speed of 51 km/h, sourced from Google Maps, was used to compute the total travel time per patient. In instances where the travel distance was 0 (for example, if a patient resided in the same postcode as the dermoscopy site), a minimum travel time of 5 min was accounted for.

**Table 2:** Calculation of distance travelled (return trips) and total clinic time per method of diagnosis

Method of diagnosis	Description	Community dermoscopy Model Distance	Face-to-face Model Distance	Community Dermoscopy Model Total Clinic Time	Face-to-face Model Total Clinic Time
A	First assessment treatment or discharge decision	cds_dist*2	hosp_dist*2	20 min	35 min
B	Required face-to-face clinic appointment	cds_dist*2 + hosp_dist*2	hosp_dist*2	50 min	35 min
C	Required diagnostic biopsy	cds_dist*2 + hosp_dist*2	hosp_dist*4	85 min	100 min
D	Required face-to-face appointment and diagnostic biopsy	cds_dist*2 + hosp_dist*4	hosp_dist*4	115 min	100 min
E	Booked for biopsy, but lesion resolved	cds_dist*2 + hosp_dist*2	hosp_dist*4	50 min	70 min

#### Clinic attendance time

The method of diagnosis determined the reported clinic durations. Waiting times due to clinic delays were not included as this data was not collected. An extra 15 min were added to account for early arrival practices for outpatient appointments in secondary care. Dermoscopy appointments typically last 20 min, while teledermatology pathway face-to-face appointments are 30 min and traditional face-to-face clinic appointments are 35 min. The shorter duration of teledermatology pathway appointments is due to clinicians having access to dermoscopy images at the start of consultation. A diagnostic biopsy procedure takes about 65 min, including wait and prep time.

#### Total time to obtain a diagnosis calculation

This value is the sum of travel time and clinic attendance time in hours.

#### Monetary cost of time calculation

To estimate monetary value of time, patients were asked to indicate their annual income bracket in the HIS. The question was structured as follows: "Q13. It would be valuable for us if you could provide us with your annual income, as income is a key determinant in healthcare (Please note this is a voluntary question). What is your estimated income per year? Please choose one of the following options:". The options ranged from 'Prefer not to say' to '£60,000 and above'. The median value of each range was then converted to an hourly wage. In cases where respondents chose 'Prefer not to say', the mean hourly wages (2022: £14.86, 2023: £12.65, or 2024: £12.02) for employed respondents or the reported market values for non-work commuting time (2022: £10.96, 2023: £10.93, or 2024: £10.94) [14] was used. Respondents with an hourly wage less than £10.93 were also adjusted to £10.93.

Monetary cost of someone's time was then multiplied by the Total Time to Obtain a Diagnosis (in hours).

#### Fuel costs calculation

The total cost of fuel was obtained by converting the distance travelled using average reported fuel consumption and price of fuel.

#### Total monetary cost calculation

The total monetary cost to obtain a diagnosis is the sum of parking costs, Monetary Cost of Time, and fuel cost.

### Statistical analyses

Student T-Tests were applied to compare the total time taken to obtain a diagnosis and the total monetary cost to obtain a diagnosis between the two cohorts (CD teledermatology and synthetic face-to-face).

### Patient and Public Involvement and Engagement (PPIE)

The patient questionnaires deployed as part of the teledermatology model, including the HIS questionnaire, were co-designed with the Open Medical PPIE committee as part of this project. This retrospective cost-analysis was borne from discussions with our PPIE committee who highlighted the importance of this research. A summary of this study was subsequently presented to the PPIE committee, who provided valuable feedback and shared their perspectives on teledermatology models used in the UK. They also discussed their experiences with digital alternatives for accessing care, which resonated with our research.

## Results

The method of diagnosis could be obtained for 1380 (out of 1384) cases, which were included in analysis. Cost calculations were performed for those patients who completed the HIS only (*n* = 619). 95.4% of patients were diagnosed through pathway A, 3.4% was diagnosed through pathway C, 0.76% through pathway B, and 0.34% through pathway D.

### Time cost

**Fig. 3:**
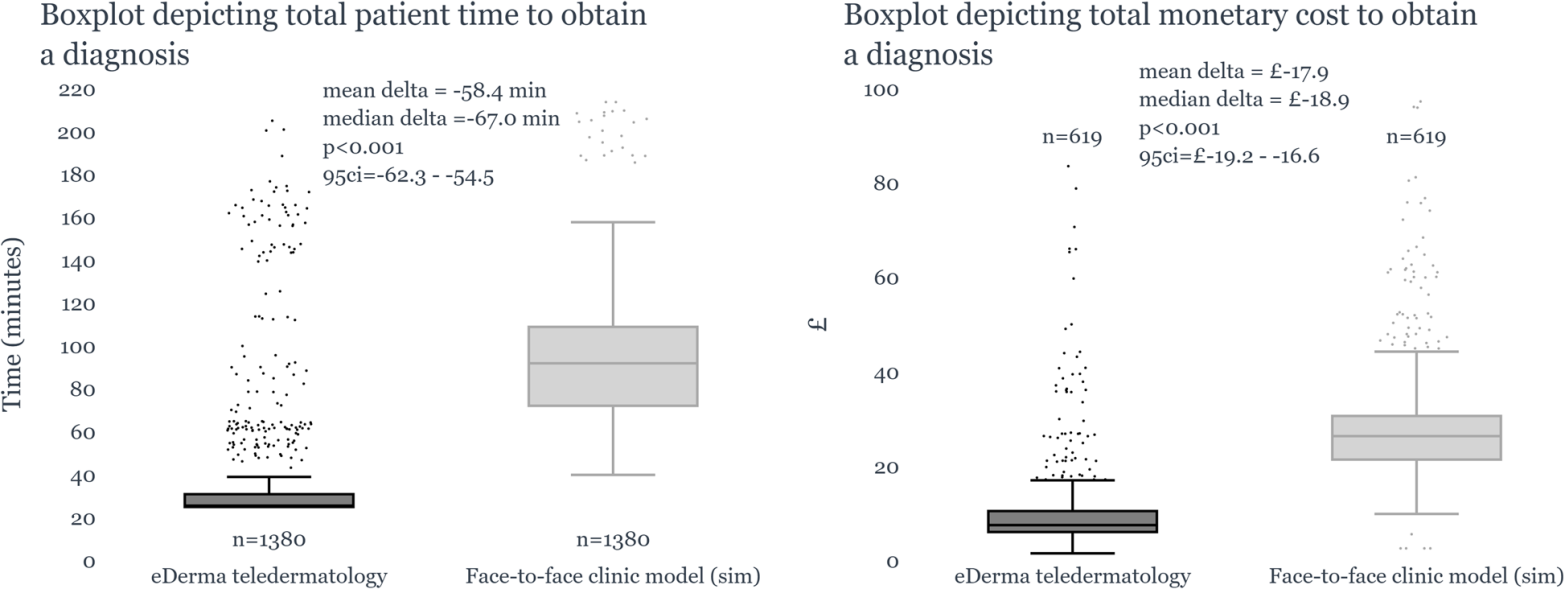
**a** (left) and **b** (right): The boxplot depicts the time taken to obtain a diagnosis (3(**a**)) and the monetary cost invested by patients (3(**b**)) in the community-based teledermatology model versus face-to-face appointments

The results obtained from our study demonstrated a significant reduction in the average time of patient involvement necessary for obtaining a diagnosis with the utilisation of the community teledermatology model. The mean time for the community-based teledermatology model was 38.1 min, in stark contrast to the time-intensive 96.7 min recorded for the traditional 'face-to-face' clinic model. This indicates a substantial time-saving of an average of 58.4 min (*p* < 0.001, 95% CI −62.3, −54.5) (Fig. 3(a)).

**Fig. 4:**
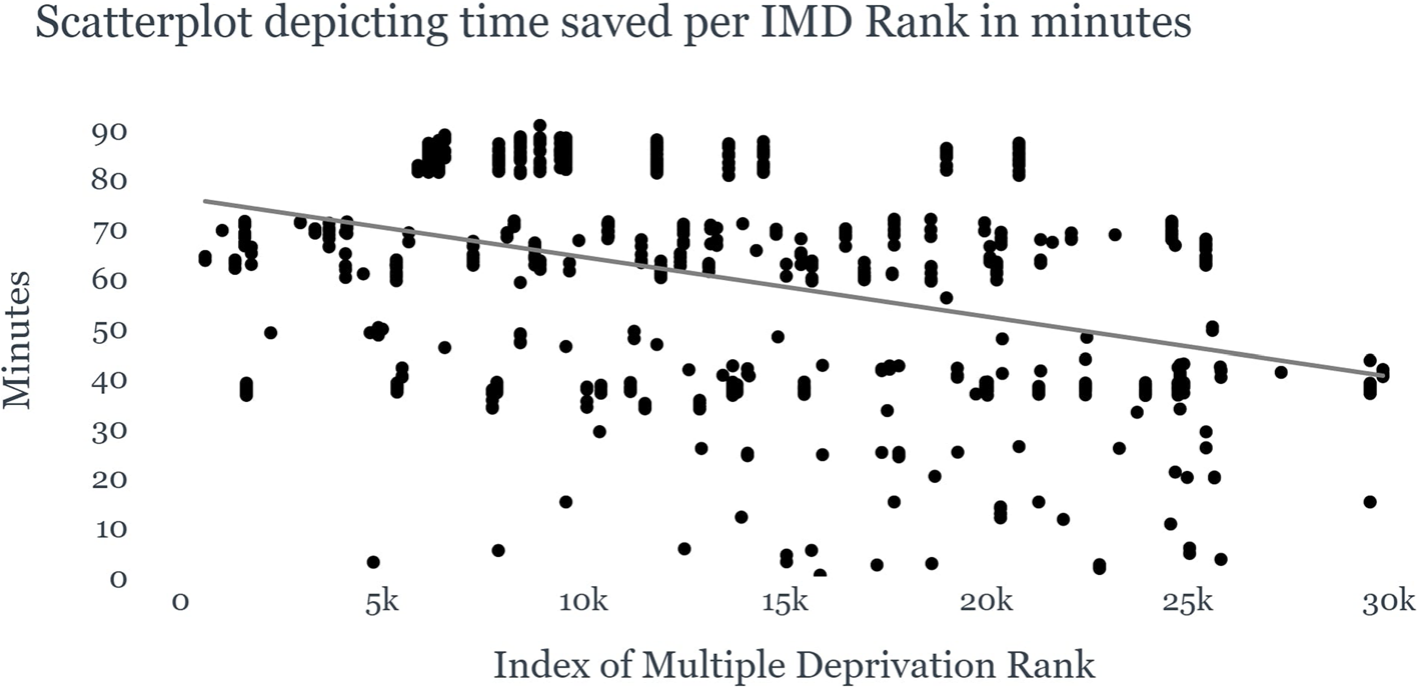
Boxplot of time-savings per IMD Rank

This time-saving was even more pronounced for patients from more deprived areas (IMDD 1–3), who saved an increased average time of 69 min compared to patients from more advantaged communities (IMDD 8–10) who saved 41.1 min on average (Fig. 4).

### Monetary cost

The community-based dermoscopy model had positive implications for patient's direct and indirect costs. These savings were realised in several areas including the reduction in parking costs, a decrease in travel distance and the associated fuel costs, and the time saved, which was translated into monetary terms.

The resulting reduction in costs was significant. On average, patients saved an estimated £17.90 per diagnosis obtained via the community-based teledermatology model, compared to the traditional face-to-face clinic model (*p* < 0.001, 95% CI -£19.2,-£16.6) (Fig. 3(b)).

Cost savings are likely to be especially impactful for patients from more deprived communities. Indeed, the majority of patients from lower IMDD regions (1–3) have an hourly wage of £10.93 or less within our analysis (whereby £10.93 is considered the lowest value-for-time considered in our model). The average hourly wage for this group is £13.28, a number heavily inflated by outliers. In contrast, the median hourly income for the high IMDD regions (8–10) is £12 and the average hourly wage is £15.6.

## Discussion

The community-based teledermatology model is more cost and time-efficient for patients than a traditional face-to-face clinic approach, particularly benefiting patients from disadvantaged backgrounds. Implementing teledermatology with community-based dermoscopy acquisition could significantly benefit regions with geographical spread and deprived communities.

Our analysis did not consider mode-of-transport in comparing different study arms. Data collected through the HIS from the teledermatology model and a face-to-face clinic arm (*n* = 37) indicated a shift from mode-of-transport from ‘Car’ to ‘On foot’, with 86.7% people travelling by car to face-to-face clinics and 60.6% travelling by car to the CD sites, while 24.7% of patients walked to the CD sites. Incorporating these figures would impact fuel and travel time calculations. However, this data was collected from a cohort that was markedly different from the study population in terms of the purpose of referrals.

Our analysis likely underestimates the real impact on patients' time. We assumed a clinic visit, including waiting time, was 35 min. In reality, waiting time can be up to an hour, making the total visit up to 3 h when travel time is included. Most patients have to take a half day off work for appointments. While teledermatology reduces appointment time to under 45 min, it's unclear if this lessens the need to take time off work. It is acknowledged that this can be particularly difficult for those in lower income jobs.

Furthermore, there are likely further delays associated with attending a face-to-face clinic appointment which we could not account for in our analysis. The EKHUFT clinical team highlights poor accessibility through public transport, frequent roadworks and congestion, and the distance of the Dermatology department from parking spaces – which can pose an obstacle for those with reduced mobility.

Lastly, we were unable to adequately account for the impact on a patient’s environment if they were a carer. While we did collect data on whether or not a patient was a carer, it is difficult to attribute a “cost” to the time lost without qualitative or in depth studies in this field.

The patient population represents cases who were referred for a skin lesion assessment on an ‘Urgent’ referral pathway, with the express purpose of excluding malignancy. However, a ‘2 week-wait’ pathway also exists for more high-risk patients. It is unclear if every patient included in our analysis would have previously received their diagnosis through a face-to-face clinic model. A subset of these patients may not have been referred for lesion assessment at all in the absence of an ‘Urgent’ pathway. The proportion of patients who would not have been managed through a face-to-face appointment in secondary care was felt to have been negligible for the purposes of this study.

The findings of this study cannot directly be applied to a ‘2 week-wait’ pathway, as it is expected that a higher proportion of patients would require face-to-face appointments or biopsy within such a pathway. Application of the statistical model with reported proportions of discharge and biopsy rates from ‘2 week-wait’ teledermatology pathways still result in time and cost-savings (ranging from 19.7 – 34.7 min and £5.2 – £11.6 on average respectively).

Our study considers the different routes a patient can undergo to obtain a diagnosis. We recognise in Pathways B and D, that teledermatology solutions may sometimes complicate a patient’s journey towards a definitive skin lesion diagnosis, as these pathways consider the necessity for a patient to have a face-to-face appointment following their remote assessment. However, real-world data derived from the remote assessment enables effective distribution of patients into these diagnostic routes.

Notably, the results of this study emphasise that initiatives that seek to provide specialist care within local communities, and those that bridge the gap between primary and secondary care, can have meaningful impacts. Procurement decisions should increasingly be made at the Integrated Care System level, with clear procurement processes that involve both primary and secondary care representatives.

## Conclusion

The community-based teledermatology model proved to be a more time and cost-efficient alternative to the traditional clinic model for patients, with the benefits amplified for patients from more deprived backgrounds. This model, implemented in an area of high coastal deprivation, offers patients a convenient way to receive care, with the option to opt for in-person consultations if desired. This flexibility improves healthcare accessibility. By providing both options, patients are also empowered to make informed decisions about the way they engage with healthcare. Importantly, the advantages in time and cost-efficiency for patients are provided at no departmental cost, given the relative cost savings of teledermatology compared to traditional face-to-face clinics [16]. These findings suggest that the wider implementation of community-based teledermatology could bring about significant benefits, particularly in areas where significant geographical spread can impact access to such services and in communities grappling with deprivation. Technologies that facilitate these community based models should integrate with existing information technology infrastructure and bridge the gap between primary and secondary care.

## Supplementary Information

Supplementary Material 1.

Supplementary Material 2.

## Data Availability

The datasets used and analysed for the current study are available from the corresponding author on reasonable request.

## Abbreviations

NHS: National Health Service
GIRFT: Get It Right First Time
EKHUFT: East Kent Hospitals University NHS Foundation Trust
CD: Community dermoscopy
HIS: Health Inequalities Survey
IMDD: Index of Multiple Deprivation Decile
PPIE: Patient and Public Involvement and Engagement
UK: United Kingdom
