# Supplementary material for: Patient cost analysis of a community-based teledermatology service versus conventional outpatient appointments in East Kent: a retrospective study through a societal lens to reduce health inequalities
Source: BMC Health Serv Res. 2024 Dec 21;24:1632. doi: 10.1186/s12913-024-12112-7 (PMC11662796; doi:10.1186/s12913-024-12112-7)
Supplement: Supplementary file 1 — Supplementary Material 1. [file 12913_2024_12112_MOESM1_ESM.pdf]

Health Inequality Survey

1. What is your primary language
   Short-answer text

2. What is your ethnicity?
   - White
   - Mixed
   - Asian/Asian British
   - Black British/Black Caribbean/Black Other
   - Arab
   - Other

3. What best describes your gender?
   - Prefer not to say
   - Male
   - Female
   - Non-binary
   - Other

4. What is your sexual orientation?
   - Heterosexual/Straight
   - Gay/Lesbian
   - Bisexual
   - Prefer not to say
   - Other

5. Do you consider yourself to have a disability?
   - Yes
   - No
   - Prefer not to say

6. If yes, does your disability impair mobility/access?
   - Yes
   - No
   - Prefer not to say

7. What is the highest level of education that you have completed?
   - High School
   - Undergraduate
   - Postgraduate
   - Other
   - No education

8. What is your employment status?
   - Employed
   - Self employed
   - Unemployed
   - Retired

CP House, 97 - 107 Uxbridge Road, London, W5 5TL, United Kingdom
www.openmedical.co.uk +44 (0)20 3475 2955

9. Your employment requires you to work outdoors?
    - Yes/no

10. What is your estimated income per year?
    - Less than £15,000
    - £15,000-£29,999
    - £30,000-£44,999
    - £45,000-£59,999
    - £60,000+
    - Prefer not to say

11. How did you travel to your appointment?
    - Car (self-driven)
    - Car (taxi)
    - Public transport
    - Bicycle
    - On foot
    - Other
