# Supplementary material for: Patient cost analysis of a community-based teledermatology service versus conventional outpatient appointments in East Kent: a retrospective study through a societal lens to reduce health inequalities
Source: BMC Health Serv Res. 2024 Dec 21;24:1632. doi: 10.1186/s12913-024-12112-7 (PMC11662796; doi:10.1186/s12913-024-12112-7)
Supplement: Supplementary file 2 — Supplementary Material 2. [file 12913_2024_12112_MOESM2_ESM.pdf]

Please complete this form before you attend your appointment.

Your answers to this questionnaire are required to help us assess the photographs of your lesions and determine the most appropriate care pathway. If you have difficulty completing the questionnaire we will be able to help with the compilation during your appointment. For more information about the teledermatology service and contact details please click the link below to access the patient information leaflet.

Consents

The consents section below allows you to choose how we use your questionnaire answers and the photographs we take of you. You can let us know if you change your mind at any time and we will update your choices.

Questionnaire consent (Please tick)

- **I consent** for the answers given in this questionnaire to be used to help assess my case.

Medical photography consent (please tick)
Images taken during your appointment will become part of your confidential medical records. Our doctors will be able to view the photographs via the hospital's electronic system. You may be asked to give consent for the photographs to be used for research and teaching. The healthcare professionals may ask for your permission to publish the photographs, for example, in a medical textbook or website. In this case you would be contacted before publication and given precise details of where the images will appear. Please select the level of consent that you wish to give.

- **I consent** for the photographs to be used anonymously for research, training, and service improvement
- **I consent** for the photographs to be used anonymously and with prior notice for publication

Assessment outcome results letter

Are you happy to receive your teledermatology assessment outcome electronically?

- **Yes**
- **No**

if "Yes" present mandatory option below

Please enter your email

Free text field

Confirm your email

© 2024 All Rights Reserved
OpenMedical

Free text field

All emails will be encrypted for security

Questionnaire

This service is for up to 2 skin lesions. Please answer the questions below (Questions 1 to 9) for the **lesion you are most worried about…**

Q1. Where is the lesion located on your body?
[Body map or just a drop down selection option of regions]

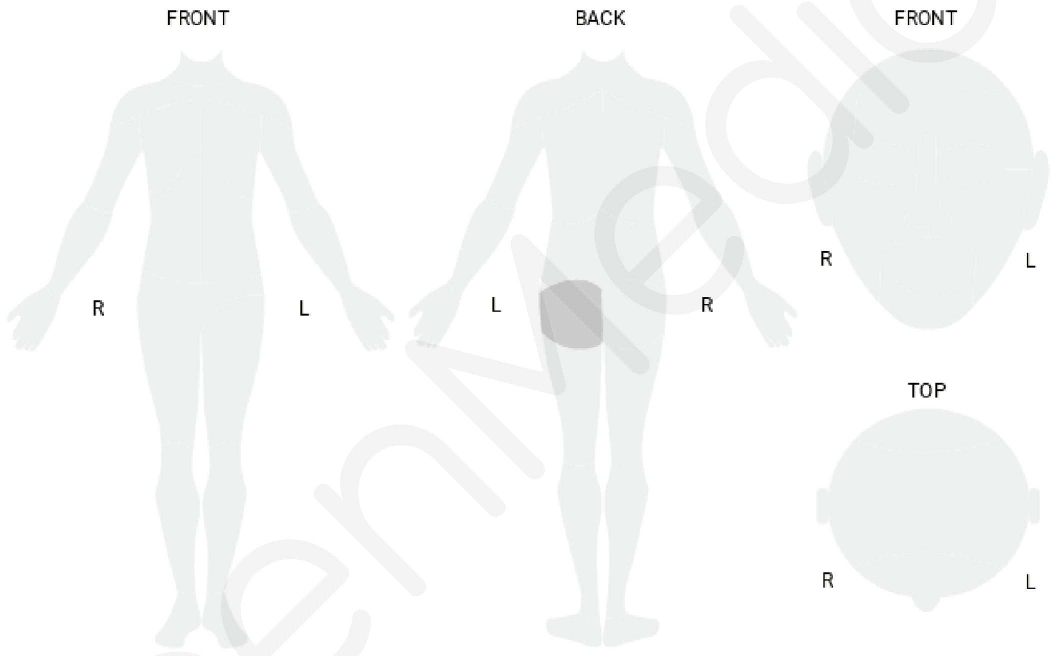

Snomed for sites

Q2. When did you first notice the lesion?

- Days
- Weeks
- Months
- Years

How many…[Patient to insert value]

Q3. When did you first notice the lesion starting to change?

- Days
- Weeks

© 2024 All Rights Reserved

- Months
- Years

How many...[Patient to insert value]

- It has not changed

Q4. Has there been any change in size?

- Bigger
- Smaller
- No change
- Not sure

Q5. Has the lesion changed in shape?

- Yes
- No
- Not sure

Q6. Has the lesion changed in colour?

- Yes
- No
- Not sure

  If "Yes" Describe

  Please describe the change in colour...

Q7. Does the lesion cause you any problems? (Tick all that apply)

- Itchy 445329008
- Bleeding 297968009
- Painful 22253000
- Other

  Specify

  Please describe other problems this lesion is causing you...

Q.8 Please add any other details you think might be helpful about the primary lesion (e.g. Has this lesion been previously treated?).

Add here any other details you wish...

Q9. How many times have you seen your GP before about **this** lesion?

- One
- Two
- Three
- Four

© 2024 All Rights Reserved

- Five
- More than five

How many lesions have you been referred for?

- One
- Two

If ***'two'*** *is selected, it should open questions 1 - 9 again for lesion 2 (Copy of EKHUFT questionnaire):*

Please answer the questions below **(Questions 1-9)** for the **second lesion**.
Q1. Where is the lesion located on your body?

Please answer the questions below (Questions 10-18) regarding your health, history, and activities.

Q10. Which of the following best describes your skin type?
If you require further information regarding this question, please access the following link:', followed by this link:
'**https://www.skinhealthinfo.org.uk/wp-content/uploads/2020/10/SKINDEX-A4-border.pdf**'

- **Always burns, never tans**
  (Pale white skin, blonde or red hair, blue eyes, freckles)
- **Usually burns, hardly tans**
  (White skin, fair complexion, blonde or red hair, blue, green or hazel eyes)
- **Sometimes mild burn, tans evenly**
  (Cream white skin, fair with any hair or eye colour)
- **Rarely burns, always tans well**
  (Moderate brown, typical Mediterranean skin tone)
- **Very rarely burns, tans very easily**
  (Dark brown skin, Middle Eastern skin types)
- **Never burns, tans very easily**
  (Deeply pigmented dark brown to black skin)

Q11. Have you previously had skin cancer?

- Yes
- No
- I don't know

If "Yes"

- Melanoma
- Squamous cell carcinoma

© 2024 All Rights Reserved

- Basal cell carcinoma
- I don't know
- Other

If "Other"

Other skin cancer

Describe the type of skin cancer...

When was it diagnosed?

Enter approximate date...

Q12. Have any <u>first degree</u> relatives ever had a mole skin cancer (melanoma)? (parent, brother, sister, child)

- Yes
- No
- I don't know

Q13. Have you ever had or do you currently have a suppressed immune system?
Caused by, for example:
- medications such as immunosuppressants (for example, *Methotrexate, Azathioprine, tacrolimus, etc)*
- a cancer treatment such as a cycle of chemotherapy and/or radiotherapy
- conditions such as certain blood cancers and organ transplants.

- Yes
- No
- Not sure

If you have answered "Yes", please provide details about the history of your suppressed immune system:

Free text field

Q14. Do you have a cardiac pacemaker or implantable electronic device?

- Yes
- No

If yes, specify which:

- Pacemaker
- Defibrillator
- Other - specify
  - FREE TEXT box

© 2024 All Rights Reserved
OpenMedical

CP House, 97 - 107 Uxbridge Road, London, W5 5TL, United Kingdom
www.openmedical.co.uk +44 (0)20 3475 2955

Q15. Do you take any blood thinning (anti-coagulants) medication? (e.g. aspirin, clopidogrel, warfarin, heparin, apixaban, rivaroxaban)

- Yes
- No

If "Yes"

- Warfarin
- Aspirin
- Rivaroxaban
- Apixaban
- Heparin
- Clopidogrel
- Other

If Yes to "Other"

Enter other blood thinning drug...

Q16. Are you pregnant or breastfeeding?

- Yes
- No
- N/A

Q17. Have you had a lot of sun exposure in the past? (e.g – lived abroad/outdoor worker/outdoor hobbies/frequent sunny holidays, use of sunbeds etc)

- Yes
- No

If answered "Yes", this opens:

Please provide details below

Free text field

Q18. Do you have any other medical conditions? (for example diabetes, heart/breathing problems, etc.)?

- Yes
- No

If "Yes"

© 2024 All Rights Reserved

CP House, 97 - 107 Uxbridge Road, London, W5 5TL, United Kingdom
www.openmedical.co.uk +44 (0)20 3475 2955

Please provide details about your medical conditions

Free text field

Q19. Which of the following would best describe your level of physical activity?

- Able to carry out all activities including strenuous activity (e.g. running)
- Able to walk and carry out light work (e.g. light housework, office work)
- Able to walk and self-care but cannot carry out work activities
- Remain in bed or chair for more that half of the day
- Remain in bed or chair for the whole day

OpenMedical

© 2024 All Rights Reserved
